# Hypotonic fluids after liver transplantation may be associated with prolonged ICU stay

**DOI:** 10.1186/cc13347

**Published:** 2014-03-17

**Authors:** A Nadeem, N Salahuddin, A ElHazmi, M Joseph, B Bohlega, H Sallam, Y Elsheikh, D Broering

**Affiliations:** 1 King Faisal Specialist Hospital & Research Centre, Riyadh, Saudi Arabia

## Introduction

Morbidity after liver transplantation has been linked with preoperative MELD scores and transfusion volumes [1]. We hypothesized that in the immediate post-transplant period there may be modifiable risk factors associated with prolonged ICU stays.

## Methods

In a retrospective, case-control study, we reviewed all liver transplant adult recipients over a 33-month period, January 2010 to September 2013. Recipients were divided into two groups based on *a priori *determined cutoff value of 8 days after transplantation. Significant associations for prolonged ICU admission were identified using chi-square analysis for categorical and ANOVA for continuous variables. *P *< 0.05 was considered significant. SPSS version 22.0 was used for all analyses.

## Results

Total numbers of transplants performed were 162. Mean pretransplant MELD score was 19.5 ± 7.7; viral hepatitis was the most common indication for transplant, 44 (27%). Living-related donor transplants were carried out in 87 (54%) cases. Median ICU length of stay was 4 ± 11.9 days and ICU mortality was 15 (9%). Acute kidney injury developed in 30 (19%) with 5% needing renal replacement therapy. Early complications developed in 33%. Prolonged ICU stay was related to: a higher pre-transplant INR (*P *= 0.000), larger volumes of RBC (2,064 ± 1,701 vs. 1,176 ± 1,454 ml, *P *= 0.000), platelets (453 ± 271 vs. 365 ± 276 ml, *P *= 0.046) and cryoprecipitate transfusions (47 ± 98 vs. 23 ± 85 ml, *P *= 0.037) received in the OR and mean volumes of hypotonic crystalloids administered in the first 24 hours (*P *= 0.002), 48 hours (*P *= 0.010) and 72 hours (*P *= 0.007) of ICU admission. Serum sodium, chloride, lactate or creatinine levels were not significantly different between the two groups.

## Conclusion

The administration of hypotonic fluids in the first 72 hours of liver transplantation may be a risk factor for prolonged ICU admission. This effect appears independent of serum electrolyte levels or renal dysfunction.

## References

[B1] Bennett-Guerrero E Preoperative and intraoperative predictors of postoperative morbidity, poor graft function, and early rejection in 190 patients undergoing liver transplantation Arch Surg 2001 136 1177 1183 10.1001/archsurg.136.10.1177 11585512

